# Simultaneous Increases in Intracellular Sodium and Tonicity Boost Antimicrobial Activity of Macrophages

**DOI:** 10.3390/cells12242816

**Published:** 2023-12-11

**Authors:** Luka Krampert, Thomas Ossner, Agnes Schröder, Valentin Schatz, Jonathan Jantsch

**Affiliations:** 1 Institute of Clinical Microbiology and Hygiene, University Hospital Regensburg and University of Regensburg, 93053 Regensburg, Germany; luka.krampert@ukr.de (L.K.);; 2 Institute of Orthodontics, University Hospital Regensburg and University of Regensburg, 93053 Regensburg, Germany; 3 Institute for Medical Microbiology, Immunology, and Hygiene, Center for Molecular Medicine Cologne (CMMC), University Hospital Cologne and Faculty of Medicine, University of Cologne, 50935 Cologne, Germany

**Keywords:** macrophages, Na^+^/K^+^-ATPase, cardiac glycosides, sodium, intracellular sodium, osmotic stress, hypertonicity

## Abstract

Inflamed and infected tissues can display increased local sodium (Na^+^) levels, which can have various effects on immune cells. In macrophages, high salt (HS) leads to a Na^+^/Ca^2+^-exchanger 1 (NCX1)-dependent increase in intracellular Na^+^ levels. This results in augmented osmoprotective signaling and enhanced proinflammatory activation, such as enhanced expression of type 2 nitric oxide synthase and antimicrobial function. In this study, the role of elevated intracellular Na^+^ levels in macrophages was investigated. Therefore, the Na^+^/K^+^-ATPase (NKA) was pharmacologically inhibited with two cardiac glycosides (CGs), ouabain (OUA) and digoxin (DIG), to raise intracellular Na^+^ without increasing extracellular Na^+^ levels. Exposure to HS conditions and treatment with both inhibitors resulted in intracellular Na^+^ accumulation and subsequent phosphorylation of p38/MAPK. The CGs had different effects on intracellular Ca^2+^ and K^+^ compared to HS stimulation. Moreover, the osmoprotective transcription factor nuclear factor of activated T cells 5 (NFAT5) was not upregulated on RNA and protein levels upon OUA and DIG treatment. Accordingly, OUA and DIG did not boost nitric oxide (NO) production and showed heterogeneous effects toward eliminating intracellular bacteria. While HS environments cause hypertonic stress and ionic perturbations, cardiac glycosides only induce the latter. Cotreatment of macrophages with OUA and non-ionic osmolyte mannitol (MAN) partially mimicked the HS-boosted antimicrobial macrophage activity. These findings suggest that intracellular Na^+^ accumulation and hypertonic stress are required but not sufficient to mimic boosted macrophage function induced by increased extracellular sodium availability.

## 1. Introduction

In addition to the renal medulla, local Na^+^ accumulation can be present in infected [1] and inflamed tissues [2,3,4,5] and can be triggered by high-salt diets [6,7]. In these microenvironments, the resulting sodium concentration can reach more than 40 mM above physiological plasma and cell culture conditions (reviewed in: [8]). These elevated Na^+^ levels influence resident and infiltrating immune cells (reviewed in: [8,9,10,11,12,13,14]). Depending on the immune cell type and subset, increased Na^+^ availability results in different activation programs and subsequent effector functions. For instance, T cells polarize towards an inflammatory Th17 phenotype under HS conditions [4,15,16,17], and regulatory T cells display a more autoimmune-like phenotype [18]. In neutrophils, short-term exposure to high Na^+^ levels can disturb the phagocyte oxidase-dependent antibacterial activity of neutrophils [19], while long-term exposure to high salt environments triggers reactive oxygen species production and proinflammatory cytokine release [20].

In macrophages, HS environments enhance proinflammatory macrophage polarization and improve their microbicidal activity (reviewed in: [8,10]). This increased macrophage activity is mediated by increased activation of osmoprotective signaling cascades, including phosphorylation of mitogen-activated protein kinase p38 (p38/MAPK) and enhanced expression of the transcription factors nuclear factor of activated T cells 5 (NFAT5) and hypoxia-inducible factor 1α (HIF1A). We recently showed that the Na^+^/Ca^2+^ exchanger 1 (NCX1) facilitates Na^+^ entry into macrophages and that NCX1-dependent Na^+^ entry is critical for mediating boosted macrophage activity and function [21,22,23,24].

The Na^+^/K^+^-ATPase (NKA) transports three Na^+^ ions from the intracellular to the extracellular space in exchange for two K^+^ ions [25,26]. The energy needed for this active transport against the concentration gradient of these electrolytes is provided by the hydrolysis of adenosine triphosphate (ATP) (reviewed in: [27]). NKA activity is known to play a key role in maintaining low intracellular Na^+^ levels and in critically contributing to the cellular membrane potential (reviewed in: [27]) [25,26]. Since NKA activity is critical for intracellular Na^+^ balance, we were interested in the effect of NKA modulation on macrophages’ activity and function. For that purpose, we resorted to pharmacological inhibition of the NKA activity with cardiac glycosides, which have been used for decades in cardiology for the treatment of arrhythmias and heart failure [28,29].

We tested whether pharmacological blockage of the NKA could mimic the responses produced by HS environments. We focused on the effects of NKA inhibition by two of the most prominent cardiac glycosides (CGs), ouabain (OUA) and digoxin (DIG). We tested if elevated intracellular Na^+^ levels caused by CG treatment under normal salt conditions could mimic the activation and function of macrophages brought about by exposure to increased extracellular Na^+^ concentrations.

## 2. Materials and Methods

### 2.1. Reagents and Antibodies

Inhibition of the NKA was performed with ouabain from Tocris (#1076) or digoxin from Sigma Aldrich (Taufkirchen, Germany) (#D6003). Macrophages were stimulated with lipopolysaccharide (LPS) from *E. coli* O111:B4 (Sigma Aldrich, #2630001M4008V). NaCl from Merck was used for HS conditions. Mannitol from Serag Wiessner (20% Mannit-solution) was used. *E. coli* HB101 [30] was kept on Mueller Hinton agar II plates, and bacterial overnight cultures were cultivated in LB media. For Western blotting, the primary antibodies rabbit anti-p38/MAPK (#8690S) and rabbit anti-phospho-p38/MAPK (#4511S) from Cell Signaling, rabbit anti-ACTIN (Sigma Aldrich, #A2066), rabbit anti-NFAT5 (Thermo Scientific, Darmstadt, Germany, #PA1-023), as well as swine anti-rabbit HRP conjugated of DAKO (#P0399) as a secondary antibody were used. Gentamicin sulfate was purchased from Sigma (#G1264).

### 2.2. Generation and Cultivation of Macrophages and Cell Lines

Bone marrow from C57BL/6 wildtype mice was used to generate bone marrow-derived macrophages (BMDMs). Animal care and use followed the regulations of the German Animal Welfare Act. Mice were housed at Zentrale Tierlaboratorien (ZTL) der Universität Regensburg and kept under conditions approved by Umweltamt der Stadt Regensburg [21]. After isolation, bone marrow cells were cultivated in Teflon bags containing L929 fibroblast supernatant, which is rich in M-CSF, as described earlier [31]. Within seven to nine days, bone marrow progenitors were differentiated into BMDMs [31]. Afterward, BMDMs were harvested and used for in vitro experiments. After seeding into cell culture plates, macrophages were incubated for at least one hour before further treatment. 

All experiments with BMDMs were performed in complete medium (CM; RPMI medium (Gibco, Darmstadt, Germany, #618700044) containing 10% fetal calf serum (FCS; Sigma Aldrich; #F7524), 50 µM 2-Mercaptoethanol (Gibco, #31350010), 10 mM HEPES (Gibco, #15630-056), and 1000 U/mL Penicillin/10 mg/mL Streptomycin (Pan Biotech, Aidenbach, Germany, #P06-07100)).

RAW264.7 cells (RAWs) are a macrophage-like immortalized cell line [32,33]. They were cultured in cell culture flasks with DMEM medium (Gibco, #41966-029) containing 10% FCS. RAWs were harvested and seeded one day before every experiment. Cells were seeded into cell culture plates at half of the desired cell number, as they were dividing during the overnight incubation (37 °C, 5% CO_2_). All experiments with RAWs were performed in CM medium.

### 2.3. Macrophage Stimulation and Infection Assay

In this study, the impact of CGs was investigated on either LPS-stimulated or *E. coli*-infected macrophages. The different experimental setups are given in Figure S1.

In the stimulation experiments, BMDMs and RAWs were stimulated with 1 ng/mL or 10 ng/mL LPS, respectively. Then, 40 mM NaCl (=high salt, HS), 80 mM mannitol (MAN), ouabain (OUA, 100 µM, unless otherwise specified), or digoxin (DIG, 50 µM, unless otherwise specified) was added as indicated.

Infection experiments were performed with BMDMs essentially as described previously [1,34]. Briefly, macrophages were seeded in 24-well plates (350,000 per well) and incubated for 1 h at 37 °C, 5% CO_2_, allowing for their attachment. Then, cells were infected with *E. coli* at a multiplicity of 100 (MOI 100), centrifuged to synchronize the infection, and subsequently treated with HS, ouabain (100 µM), digoxin (50 µM), or mannitol as indicated. At 1 h post-infection, the macrophages were washed twice with PBS, gentamicin (100 µg/mL) was added to eliminate extracellular bacteria, and cells were treated with HS, mannitol, and cardiac glycosides and incubated for another hour. Afterward, cells were washed with PBS and lysed with 0.1% Triton/0.05% Tween-20 in PBS, and then serial dilutions were plated on Mueller Hinton agar plates. On the next day, colony-forming units (CFUs) were counted and normalized to the mean of the respective control group.

### 2.4. E. coli Growth Curves

Bacterial growth curves were analyzed to determine the potential influence of different stimulations and compounds on the growth properties of *E. coli* HB101. Liquid *E. coli* overnight cultures were adjusted to an OD_600_ value of 0.1. Bacterial growth was monitored by measuring OD_600_ values over time in the absence or presence of cardiac glycosides at 37 °C and an atmosphere of 5% CO_2_.

### 2.5. Quantification of Nitrite Concentration and Lactate Dehydrogenase Activity

Griess assays of the cell supernatants were performed 24 h after stimulation to determine the nitrite (NO_2_^−^) concentrations. Nitrite levels were measured as described previously [1,34].

Cell cytotoxicity was determined by measuring lactate dehydrogenase (LDH) activity with a cell cytotoxicity kit (Roche; #11644793001) as described earlier [21]. LDH activity was determined in cell supernatants and lysates according to the manufacturer’s instructions. The ratio of supernatant to pellet was calculated as a measure of cellular viability. Cells treated with 0.1% Triton X-100 30 min before lysis were used as a positive control for cell death.

### 2.6. Immunoblotting

Western blotting was performed to determine the expression of phospho-p38/MAP kinase (p-p38/MAPK) and NFAT5. RAWs were seeded into 6-well plates with a concentration of 1 × 10^6^ cells/mL one day before the experiment and incubated overnight (37 °C, 5% CO_2_). The next day, the cells were stimulated with LPS (10 ng/mL) and simultaneously treated as indicated. After specific time points (p-p38/MAPK: 45 min; NFAT5: 24 h), the cells were lysed in either RIPA buffer (25 mM sodium deoxycholate, 1% SDS, 0.4% EDTA, 10 mM NaF, 1% NP 40 in H_2_O_dd_) containing complete protease inhibitors (Roche, Taufkirchen, Germany, #1183617001) and PhosSTOP^TM^ (Roche, #04906837001) to determine pp38/MAPK abundance, or in 8 M urea containing complete protease inhibitors (Roche, #1183617001) for NFAT5 analysis. Immunoblotting was performed as described earlier [1,34]. Briefly, cell lysates were homogenized, then protein concentrations were determined with the DC™ protein assay (BioRad, Kabelsketal, Germany), and proteins were separated on a 12% (pp38/MAPK) or 8% (NFAT5) TRIS-glycine polyacrylamide gel. Then, the proteins were transferred to a PVDF membrane (Merck, Molsheim, France, IPFL 00010). Signals were detected on a Chemo Star imager (Intas) using Luminata Forte HRP substrate (Millipore, Molsheim, France, #WBLUF0500). 

### 2.7. Measurement of Intracellular Na^+^ and K^+^ Levels with Atomic Absorption Spectrometry (AAS)

Intracellular Na^+^ and K^+^ levels in cell lysates were measured with a flame atomic absorption spectrometer (iCE3500 AA system, Thermo Scientific) essentially as described earlier [35]. For that purpose, cells were seeded into 12-well plates (1 × 10^6^ cells/well), stimulated with LPS (BMDMs: 1 ng/mL; RAWs: 10 ng/mL), and treated as indicated. After indicated time points, cells were washed three times with an iso-osmolal sucrose solution (Ctrl, OUA, DIG: 290 mOsmol/mL; HS: 350 mOsmol/mL) and lysed with Na^+^-free cell lysis buffer III (0.1% Triton X-100 in H_2_O_dd_). The AAS was calibrated with a series of commercial Na^+^ or K^+^ AAS standard solutions (Carl Roth, Na^+^: #2337.2; 0.075–0.5 mg/L; potassium: #2327.2; 0.1–1.0 mg/L) before the measurements. The correlation coefficients for the linear calibration curve were >0.995. Ion concentrations of the samples were determined and normalized to the mean of the respective control group.

### 2.8. Rubidium Incorporation Measurements with Atomic Absorption Spectrometry

In order to assess the inhibition of the NKA with the cardiac glycosides, rubidium (Rb^+^) incorporation assays were performed following a protocol published by Gill et al. [36]. Rb^+^ can be used as a non-radioactive K^+^ tracer. For these assays, the RAWs and BMDMs were seeded into 12-well plates (1 × 10^6^ cells/well) and incubated for 1 h at 37 °C, 5% CO_2_, allowing their attachment. Then, the medium was replaced by Rb^+^ uptake buffer (15 mM HEPES, 140 mM NaCl, 5.4 mM RbCl, 1 mM MgCl_2_, 0.8 mM NaH_2_PO_4_, 2 mM CaCl_2_, pH 7.4). Macrophages were stimulated with LPS (BMDMs: 1 ng/mL; RAWs: 10 ng/mL) and simultaneously treated with the CGs as indicated. After indicated time points, the cells were washed three times with an iso-osmolal sucrose solution (Ctrl, OUA, DIG: 290 mOsmol/mL) and lysed with lysis buffer III (0.1% Triton X-100 in H_2_O_dd_). The AAS was calibrated with a series of Rb^+^ standard solutions (Carl Roth, #2456.1; 0.063–16 mg/L) before the measurements. The correlation coefficients for the linear calibration curve were routinely >0.995. Ion concentrations of the samples were determined and normalized to the mean of the respective control group.

### 2.9. Determination of Intracellular Ca^2+^-Levels with Epifluorescence Microscopy

Calcium (Ca^2+^) levels were measured by epifluorescence microscopy using the Ca^2+^-sensitive dye Fura-2 (Thermo Scientific, #F1221) as described earlier [21]. BMDMs were seeded on FluoroDish plates (2 × 10^6^ cells/dish) and stained with Fura-2 in Tyrode solution (140 mM NaCl, 4 mM KCl, 1 mM MgCl_2_, 5 mM HEPES, 1 mM CaCl_2_, and 10 mM glucose) containing 0.04% Pluronic (Sigma, #P2443). Fura-2-loaded cells were analyzed via live cell imaging using epifluorescence microscopy (Motic model 410E). After 10 s of baseline detection, the cells were stimulated with LPS (1 ng/mL) and simultaneously treated as indicated. Fura-2-excitation was detected every 30 s for 7 min. After subtraction of the background signals, the relative [Ca^2+^]_i_ levels were determined by ratiometric quantification and normalized to baseline signals before treatment.

### 2.10. IL-1ß Quantification via Enzyme-Linked Immunosorbent Assay

IL-1β secretion was determined by an enzyme-linked immunosorbent assay (ELISA). Therefore, 0.8 × 10^6^ BMDMs/mL were seeded into 12-well plates and incubated overnight (37 °C, 5% CO_2_). On the following day, macrophages were primed with high concentrations of LPS (1 µg/mL) for 4 h (37 °C, 5% CO_2_). After 4 h, the BMDMs were treated as indicated and incubated for 2 h (37 °C, 5% CO_2_) before supernatants were collected. IL-1β levels in the supernatants were measured with the mouse IL-1 beta/IL-1F2 DuoSet ELISA kit (R&D Systems, Wiesbaden, Germany, #Dy401) according to the manufacturer’s instructions. Signals were developed with the BD OptEIA™ TMB substrates. The reaction was stopped with 50 µL 1 M HCl, and the absorption was measured at 450 nm with a microplate reader.

### 2.11. RNA Isolation, Reverse Transcription, Real-Time PCR, and Relative Quantification

RNA isolation and subsequent qRT-PCR were performed as described earlier [34]. Briefly, RNA was isolated with RNA Solv Reagenz^®^ (VWR, #R6830-01), isopropanol, and 70% ethanol. The isolated RNA was transcribed into cDNA by reverse transcription with the High-Capacity cDNA Reverse Transcription Kit (Applied Biosciences, Darmstadt, Germany, #4368813). For the qRT-PCR, the TaqMan^®^ probes for *Hprt* (Mm00446968_m1) and *Nfat5* (Mm00467257_m1) from Thermo Scientific were used. For relative quantification, a ΔΔCT-ratio between the gene of interest (*Nfat5*) and the endogenous control gene (*Hprt*) was calculated. Ratios were normalized to control conditions.

### 2.12. Statistical Analyses

For graph design and statistical analyses of generated data sets, the GraphPad Prism was used (v 8.0). First, the normal distribution was analyzed in all datasets with a Kolmogorov–Smirnov normality test. Datasets with equal distribution in every group were analyzed with an unpaired *t*-test comparing the two groups. When needed, Welch’s correction was performed. When more than two groups were analyzed, an ordinary one-way ANOVA with Bonferroni’s post hoc test was performed. If at least one group was unequally distributed, a Mann-Whitney test was used for the comparison of two groups. For multiple comparisons, a Kruskal-Wallis test with Dunn’s multiple comparisons test was performed. As AAS detects minimal ion concentrations, we used the ROUT outlier test with an excluding criterion of 1% to identify outliers, as described earlier [37]. Densitometric evaluation of immunoblotting data was analyzed with a paired *t*-test. Finally, a two-way ANOVA with Geisser–Greenhouse correction was used for time-dependent measurements like intracellular Ca^2+^ levels and bacterial growth curves. The mean of all data groups and the standard error of the mean (s.e.m.) are shown in the graphical presentations. Within all tests, *p* values < 0.05 were considered statistically significant.

## 3. Results

High Na^+^ environments trigger increases in intracellular Na^+^ and boost the inflammatory and antimicrobial activity of macrophages [1,21,34,38]. NKA activity plays a key role in the maintenance of intracellular ion balance since it catalyzes the efflux of three Na^+^ ions from the intracellular space in exchange for two K^+^ ions to the intracellular space [25,26,39] (reviewed in: [27]). Here, we investigated whether the pharmacological blockage of the NKA results in a similar increase in intracellular Na^+^ and a similar boost of a proinflammatory phenotype of macrophages. Therefore, BMDMs and RAWs (hatched graphs) were treated with two cardiac glycosides, OUA and DIG, to investigate if effects induced by high salt treatment (HS; +40 mM) could be mimicked.

### 3.1. CGs Induce Intracellular Na^+^ Accumulation Similar to HS Conditions

NKA catalyzes the efflux of three Na^+^ from the cytosol in exchange for two K^+^ into the intracellular space [39]. In the first step, therefore, concentrations were determined for the two NKA inhibitors at which there is an intracellular Na^+^ (Figure S2) increase and a simultaneous drop in intracellular K^+^ levels (Figure S3) in BMDM and RAWs (hatched graphs). The 50 µM DIG and 100 µM OUA fulfilled these requirements. (Figures S2 and S3). An Rb^+^-uptake assay was performed to corroborate these findings with the chosen inhibitor concentrations. In this assay, extracellular K^+^ is substituted by Rb^+^, which can be separated from K^+^ in the chemical analysis. Exposure of macrophages to the selected CG concentrations decreased the Rb^+^-uptake, confirming the successful NKA inhibition (Figure S4).

**Figure 1:**
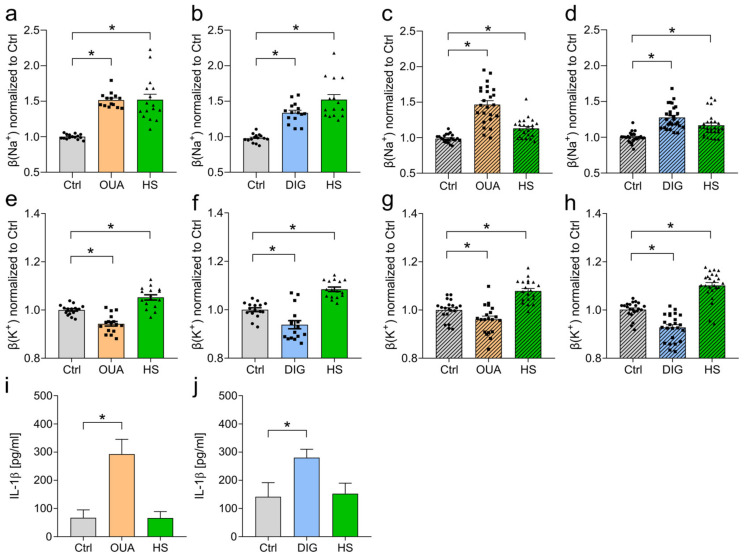
Pharmacological Na^+^/K^+^-ATPase inhibition results in elevated intracellular Na^+^ levels but a loss of K^+^ and subsequent IL-1ß release. (**a**,**b**) BMDMs were stimulated with LPS and (**a**) ouabain (OUA), (**b**) digoxin (DIG), or NaCl (HS). [Na^+^]_i_ levels normalized to control conditions (Ctrl) were determined (means ± s.e.m; n = 14–16; ordinary one-way ANOVA with Bonferroni’s multiple comparisons tests; * *p* < 0.05). (**c**,**d**) As in (**a**,**b**), but RAWs (hatched graphs) were used (means ± s.e.m; n = 23–28; ordinary one-way ANOVA with Bonferroni’s post hoc test and Kruskal–Wallis test with Dunn’s multiple comparisons test; * *p* < 0.05). (**e**,**f**) As in (**a**,**b**), but [K^+^]_i_ levels normalized to Ctrl were determined (means ± s.e.m; n = 16; ordinary one-way ANOVA with Bonferroni’s multiple comparisons tests; * *p* < 0.05). (**g**,**h**) As in (**c**,**d**), but [K^+^]_i_ levels normalized to Ctrl were determined (means ± s.e.m; n = 20–24; ordinary one-way ANOVA with Bonferroni’s multiple comparisons test and Kruskal–Wallis test with Dunn’s multiple comparisons test; * *p* < 0.05). (**i**,**j**) BMDMs were primed with 1 µg/mL LPS and treated with (**i**) OUA, (**j**) DIG, or HS. IL-1β levels in supernatants were determined (means ± s.e.m; n = 12; Kruskal–Wallis test with Dunn’s multiple comparisons test; * *p* < 0.05).

Next, we treated the BMDMs (Figure 1a,b) and RAWs (Figure 1c,d) with LPS and simultaneously with previously determined CG concentrations (Figures S2 and S3) or HS. In both cell types, inhibition of the NKA with OUA (Figure 1a,c) and DIG (Figure 1b,d) resulted in a similar Na^+^ accumulation in the intracellular space as under HS conditions.

### 3.2. Loss of Potassium and Increase in IL-1β Production upon CG but Not HS Treatment 

Using the inhibitory concentrations, we determined intracellular K^+^ levels in macrophages treated with OUA or DIG and compared them to K^+^ concentrations of HS-exposed cells. Again, the BMDMs and RAWs were stimulated with LPS and simultaneously treated with CGs or HS. Here, we observed the first differences between the CG and HS treatment. In contrast to HS conditions, the OUA and DIG treatments led to a loss of intracellular K^+^ in the BMDMs (Figure 1e,f) and RAWs (Figure 1g,h).

Loss of intracellular K^+^ is an inflammasome activation signal [40,41,42,43]. The assembly of NOD-like receptor-pyrin-containing proteins 3 (NLPR3) and other associated proteins such as apoptosis-associated speck-like proteins (ASC) to a hetero-oligomeric complex activates caspase 1 (reviewed in: [44]). Upon this activation, pro-IL-1β is cleaved by proteolysis, and proinflammatory IL-1β is secreted (reviewed in: [45]). In line with the loss of potassium after cardiac glycoside treatment, the BMDMs released significantly higher amounts of IL-1β (Figure 1i,j). In contrast, macrophages exposed to HS conditions accumulated intracellular K^+^ and did not produce higher levels of IL-1β (Figure 1i,j).

### 3.3. Cardiac Glycosides Do Not Influence Intracellular Calcium Levels

**Figure 2:**
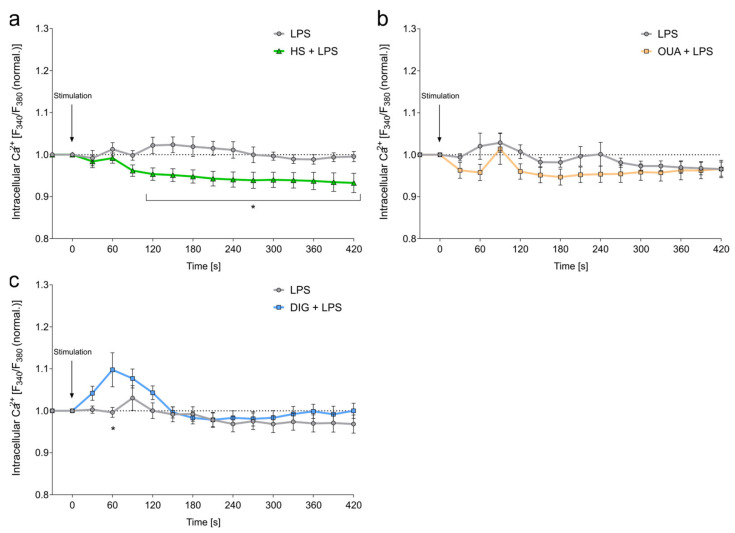
Cardiac glycosides do not diminish intracellular Ca^2+^ levels. (**a**–**c**) Fura-2-loaded BMDMs were stimulated with LPS and simultaneously treated with (**a**) HS, (**b**) OUA, or (**c**) DIG for 7 min, and the relative [Ca^2+^]_i_ levels were recorded. Data were normalized to baseline signals before treatment (means ± s.e.m; n = 8–10; two-way ANOVA with Geisser–Greenhouse correction; * *p* < 0.05).

Upon HS exposure, intracellular Ca^2+^ and Na^+^ levels are tightly intertwined [21,46]. Moreover, Ca^2+^ can play an important role in the activation and signaling of macrophages [47]. With epifluorescence microscopy, intracellular Ca^2+^ levels were measured after stimulation with LPS and treatment with OUA, DIG, or HS. In line with earlier findings [21], HS exposure diminished intracellular Ca^2+^ levels. They were lower from 120 s after stimulation. (Figure 2a). In contrast, this finding was not recorded after exposure of cells with CGs (Figure 2b,c). OUA treatment slightly reduced intracellular Ca^2+^ levels but returned to control levels afterward, whereas the DIG treatment showed a transient early Ca^2+^ peak after 60 s.

### 3.4. Cardiac Glycosides Induce p-p38/MAPK Signaling, Whereas NFAT5 Expression Remains Unchanged

**Figure 3:**
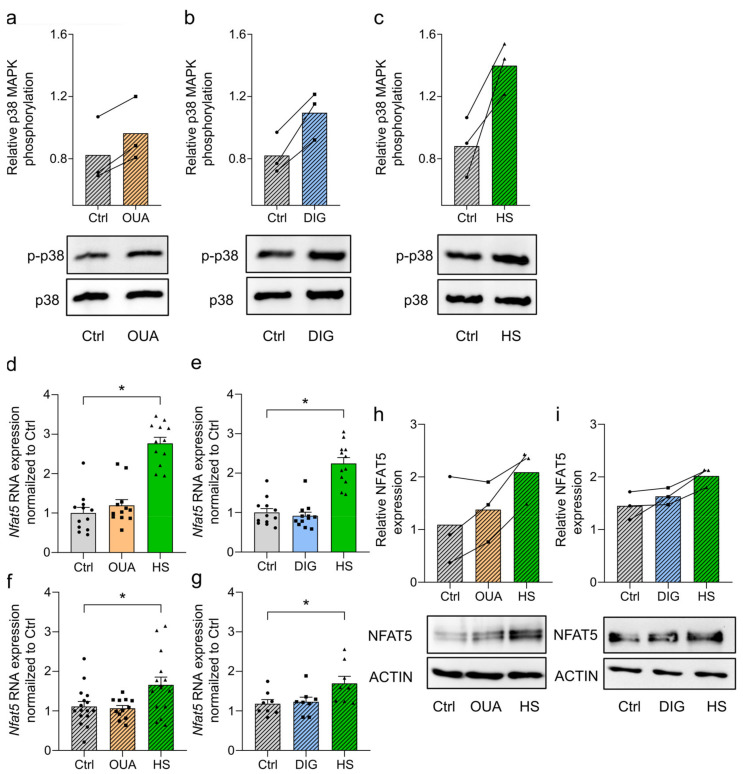
Cardiac glycosides induce phosphorylation of p38/MAPK, whereas NFAT5 expression remains unchanged. (**a**–**c**) LPS-stimulated RAWs were treated with (**a**) OUA, (**b**) DIG, or (**c**) HS. p38/MAPK phosphorylation was determined (n = 3). Representative blots for each condition are shown. (**d**,**e**) *E. coli*-infected BMDMs were treated with (**d**) OUA, (**e**) DIG, or HS. After 2 h, relative *Nfat5* RNA expression (to Ctrl) was determined (means ± s.e.m; n = 12; Kruskal–Wallis test with Dunn’s multiple comparisons test; * *p* < 0.05). (**f**,**g**) As in (**d**,**e**), RAWs were used (means ± s.e.m; n = 8–16; ordinary one-way ANOVA with Bonferroni’s multiple comparisons test; * *p* < 0.05). (**h**,**i**) RAWs were stimulated with LPS, and (**h**) OUA, (**i**) DIG, or HS. NFAT5 protein levels were detected after 24 h (n = 3). Representative blots are displayed.

After the electrolyte measurements, we investigated CGs’ impact on osmotic stress response pathways. Upon HS exposure, macrophages phosphorylate p38/MAPK as an osmotic stress response [1,48]. RAWs were stimulated with LPS together with OUA, DIG, or HS for 45 min. OUA (Figure 3a) and DIG (Figure 3b) treatments resulted in the phosphorylation of p38/MAPK on protein levels, similar to HS conditions (Figure 3c).

Then, we analyzed the expression of the osmoprotective transcription factor NFAT5 because it is a downstream target of p-p38/MAPK and has a central role in sodium-boosted, proinflammatory macrophage function [1,34]. During infection with *E. coli*, macrophages did not induce *Nfat5* RNA expression upon cardiac glycoside treatment (Figure 3d–g). At protein levels after 24 h of LPS stimulation, HS conditions resulted in a significant induction of NFAT5. OUA and DIG, however, did not induce NFAT5 expression reliably (Figure 3h,i). Despite the intracellular Na^+^ accumulation, macrophages did not upregulate *Nfat5* RNA or NFAT5 protein expression after OUA and DIG treatment.

### 3.5. Heterogeneous Effects of CGs and HS towards NO Production and the Elimination of Intracellular E. coli

NFAT5 regulates the transcription of different genes, for example, the type 2 nitric oxide synthase (*Nos2*) [1,49,50] (reviewed in: [51]). This enzyme catalyzes the production of NO from the amino acid L-arginine. Especially in macrophages, NO production is a central effector function in eliminating invading pathogens (reviewed in: [52,53]).

**Figure 4:**
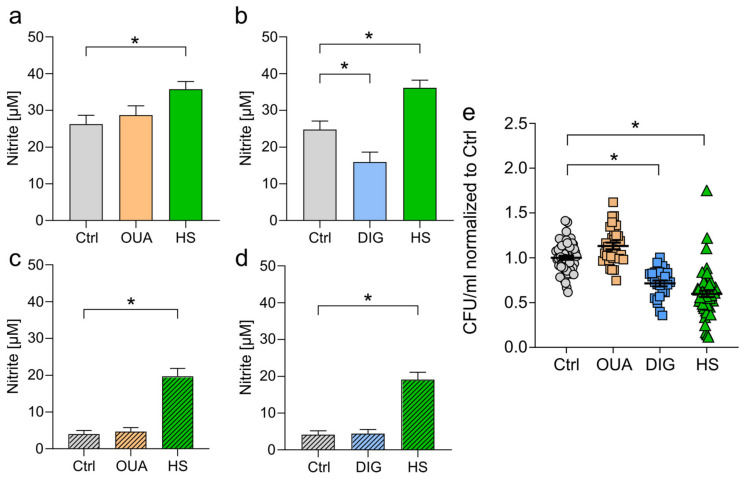
Divergent effects of CGs and HS on NO production and the elimination of intracellular *E. coli*. (**a**,**b**) LPS-stimulated BMDMs were treated with (**a**) OUA, (**b**) DIG, or HS. After 24 h, nitrite levels were determined in supernatants (means ± s.e.m; n = 30; Kruskal–Wallis test with Dunn’s multiple comparisons test; * *p* < 0.05). (**c**,**d**) As in (**a**,**b**), RAWs were used (means ± s.e.m; n = 20; Kruskal–Wallis test with Dunn’s multiple comparisons test; * *p* < 0.05). (**e**) BMDMs were infected with *E. coli* and treated with OUA, DIG, or HS. Intracellular bacterial load (normalized to Ctrl) was determined (means ± s.e.m; n = 25–55; Kruskal–Wallis test with Dunn’s multiple comparisons test; * *p* < 0.05).

In line with the unchanged NFAT5 expression upon CG exposure, *Nos2*-dependent NO production was not induced in BMDMs (Figure 4a,b) and RAWs (Figure 4c,d). Nitrite levels were even lower after DIG exposure in BMDMs (Figure 4b). In contrast, HS treatment boosted NO production in both cell types after 24 h of LPS stimulation (Figure 4a–d), in line with earlier findings [1,49,50]. Furthermore, neither HS nor OUA or DIG treatment had cytotoxic effects on the BMDMs (Figure S5a,b) and RAWs (Figure S5c,d).

In addition, macrophages can phagocytose and directly eliminate bacteria and other pathogens. Macrophages recognize bacterial invaders by receptors, internalize them, and digest them in (auto)lysosomal compartments [54]. We infected BMDMs with *E. coli* and determined intracellular bacterial numbers after two hours. OUA treatment had no significant impact on the bacterial load, whereas DIG treatment favored the clearance of *E. coli* (Figure 4e). Therefore, cardiac glycoside treatment had heterogeneous effects on the elimination of intracellular bacteria. In line with previously published data [19,34,35], macrophages exposed to HS conditions displayed an increased antibacterial activity, resulting in a lower bacterial load (Figure 4e). Treatment with OUA, DIG, or HS did not impair the growth of *E. coli* (Figure S5e,f).

### 3.6. Intracellular Na^+^ Accumulation and Hypertonic Stress Increase NFAT5 Expression and Antibacterial Activity

Based on these findings, we concluded that intracellular Na^+^ accumulation alone is not sufficient to mimic the previously described sodium-boosted antimicrobial macrophage function. In contrast to cardiac glycosides, HS conditions, however, induce hypertonic stress in addition to increases in intracellular Na^+^ levels. This is why we sought to increase tonicity together with CG treatment to mimic HS conditions better. We, therefore, treated macrophages with the non-ionic osmolyte mannitol (MAN; 80 mM; iso-osmolar to HS conditions) in combination with OUA. MAN is known to induce hypertonic stress in macrophages [55,56] without inducing intracellular Na^+^ accumulation (Figure S5g) [34].

**Figure 5:**
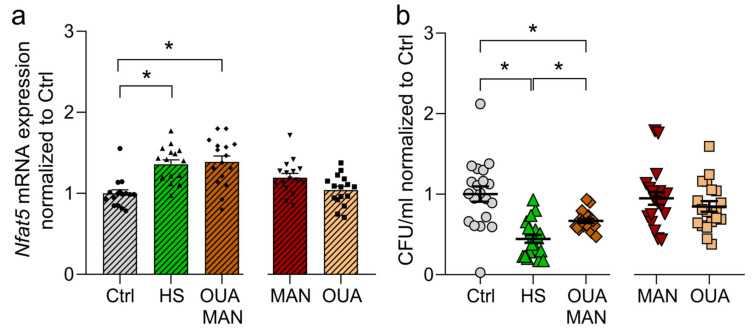
Combination of mannitol and ouabain boosts NFAT5 expression and the antibacterial activity of macrophages. (**a**) LPS-stimulated RAWs were treated with HS, MAN, OUA, or a combination of MAN and OUA. After 4 h, relative *Nfat5* mRNA expression (normalized to Ctrl) was determined (means ± s.e.m; n = 16; Kruskal–Wallis test with Dunn’s multiple comparisons test; * *p* < 0.05). (**b**) BMDMs were infected with *E. coli* and treated with HS, MAN, OUA, or a combination of MAN and OUA. Intracellular bacterial load (normalized to Ctrl) was determined (means ± s.e.m; n = 20; ordinary one-way ANOVA with Bonferroni’s multiple comparisons test; * *p* < 0.05).

Since enhanced NFAT5 expression is a key feature of HS-boosted proinflammatory macrophage activity, we analyzed the impact of the combination treatment of MAN and OUA on NFAT5 expression. Cotreatment of MAN and OUA led to a significant increase in *Nfat5* mRNA expression, similar to HS conditions (Figure 5a), without inducing cytotoxicity (Figure S5h).

We have shown earlier that HS-enhanced antibacterial activity critically requires NFAT5 expression for HS-boosted antibacterial activity [34]. Since MAN, together with OUA, increased NFAT5 levels, we sought to analyze the impact on macrophages’ bactericidal activity. We observed an improved antibacterial activity of macrophages when treated with the combination of MAN and OUA, while no effect was observed with MAN or OUA alone (Figure 5b). However, high Na^+^ environments induce a stronger antibacterial boost (Figure 5b). Thus, MAN and OUA were partially able to resemble the HS-boosted antibacterial phenotype of macrophages. Overall, we conclude that increases in intracellular Na^+^ levels are not sufficient to mimic HS-boosted antimicrobial activity but require, in addition, other signals, such as hypertonic stress.

## 4. Discussion

The role of elevated intracellular Na^+^ levels on immune cell function has been studied extensively over the last few years (reviewed in: [8,9,10,11,12,13,14]). HS environments modulate immune cell activity and function [1,18,19,34,35]. Increases in extracellular Na^+^ can trigger increases in intracellular Na^+^ levels [18,21,35], which are linked to altered immunobiology of macrophages and T cells. Here, we investigated if raising intracellular Na^+^ by NKA inhibition is sufficient to mimic the HS-boosted proinflammatory activity of macrophages. All our experiments were performed either in the RAW 264.7 macrophage cell line or in primary bone marrow-derived macrophages. As both types of macrophages display the same HS-triggered proinflammatory and boosted antimicrobial phenotype [1,34], we used them interchangeably in this study.

The role of the NKA as an ion-independent signal transducer has been established [57,58]. With its signaling function, the NKA is involved in various physiological and pathophysiological processes like regulation of blood pressure, natriuretic control in the kidney, and heart function (reviewed in: [27]). Moreover, NKA inhibition by cardiac glycosides influences the activation and function of immune cells (reviewed in: [59]). For instance, cardiac glycosides are capable of influencing the function of immune cells in a dose-dependent manner. While high levels of DIG have been shown to inhibit T_H_17 differentiation via RORγT [60], DIG at lower concentrations has been described to be an agonist for RORγT [61]. In our study, we applied OUA and DIG in the micromolar range to impair the ionic transport activity of the NKA. We sought to induce intracellular Na^+^ accumulation rather than addressing potential ion transport-independent signaling functions of the NKA.

In line with earlier findings [21,35], we observed increased intracellular Na^+^ levels in macrophages under HS conditions. Inhibition of the NKA with OUA and DIG displayed a similar effect. While elevated intracellular Na^+^ levels were accompanied by an intracellular K^+^ increase upon HS exposure, pharmacological blockage of the NKA caused an expected intracellular K^+^ loss. As described elsewhere [43,62], this was followed by inflammasome activation and increased IL-1β production. In line with elevated intracellular K^+^ concentrations, IL-1β levels remained low under HS conditions. In contrast, Ip and Medzhitov showed ROS-dependent inflammasome activation under hyperosmotic conditions [63]. However, they applied higher Na^+^ concentrations (+100 mM) in comparison to our HS condition (+40 mM), and their inflammasome activation was triggered in an NKA-independent way [63].

In cardiomyocytes [29], CGs have been shown to induce intracellular Na^+^ accumulation, thereby attenuating NCX1-driven Ca^2+^ export [64,65,66]. Apart from a small Ca^2+^ peak early after DIG treatment, both CGs had no short-term impact on the intracellular Ca^2+^ levels in macrophages. This could be due to the different resting membrane potentials of cardiomyocytes and macrophages, which could subsequently have a different impact on the thermodynamics of the NCX activity. Macrophages have a resting membrane potential between −20 and −40 mV [21,67,68,69], while cardiomyocytes’ resting membrane potential is around −90 mV [70]. NCX mode of action depends on the membrane potential [21,22]. In addition, cardiomyocytes have commonly expressed Ca^2+^-transporters and intracellular Ca^2+^-stores that are activated upon OUA treatment [71]. Moreover, they also have other specific ion channels [72]. This clearly distinguishes their Ca^2+^-signaling from that of other cells, such as macrophages.

In macrophages, HS environments enhanced p38/MAPK phosphorylation, subsequent expression of the osmoprotective transcription factor NFAT5, and NO release [1,34,48,73,74,75] (reviewed in: [8,10]). Treatment with both CGs led to a comparable induction of p-p38/MAPK but did not affect NFAT5 on RNA or protein levels. Additionally, cells treated with cardiac glycosides did not upregulate NO production. On the contrary, DIG-treated macrophages even displayed an impaired NO release. This indicates that CG-induced p38/MAPK activation does not trigger subsequent NFAT5-dependent activation, which is critical for osmoprotective HS-triggered macrophage responses. 

The effect of CGs on p38/MAPK phosphorylation has been shown to be cell-type-dependent. OUA treatment has been described to inhibit p38/MAPK activation in thymocytes [76] and neutrophils [77] but to induce p38/MAPK phosphorylation in human monocytes [78]. Since HS conditions trigger p38/MAPK activation and increased Na^+^ levels in T cells [15,18] and macrophages [1,21], it is possible that CG treatment-induced p38/MAPK activation is not due to enhanced intracellular Na^+^-levels but triggered by other CG-induced signaling events.

High Na^+^ environments lead to intracellular Na^+^ accumulation and also cause a hypertonic environment, affecting cellular physiology. Treatment with the two cardiac glycosides OUA and DIG only induces the first. Hence, we hypothesized that Na^+^ accumulation alone is not sufficient to mimic the observed HS phenotype in macrophages. We adjusted our experimental setup accordingly. We sought to mimic HS conditions better by a combination treatment of OUA and MAN, a non-ionic osmolyte. MAN has been shown to be a potent inducer of hypertonic stress [55,56,79]. Indeed, we were able to induce *Nfat5* mRNA expression similar to HS conditions when treating macrophages with the combination of MAN and OUA. The combination of both also improved the killing capacity of macrophages significantly. However, we could not completely mimic the condition induced by high salt. Among other hitherto unidentified factors requiring further investigation, intracellular Na^+^ availability and enhanced tonicity play a crucial role in the induction of HS-enhanced macrophage activity.

## 5. Conclusions

HS environments trigger an NCX1-dependent entry of Na^+^, which subsequently boosts the proinflammatory activation of macrophages by p38/MAPK signaling and downstream NFAT5 expression [1,34]. In this report, we asked whether intracellular Na^+^ accumulation is sufficient to cause this proinflammatory macrophage phenotype. Therefore, we blocked the NKA pharmacologically to increase intracellular Na^+^ levels similar to those under HS conditions. We detected a similar intracellular Na^+^ build-up with the two cardiac glycosides, OUA and DIG. We observed similarities between macrophages exposed to HS environments or CGs. However, we were only partially able to mimic the proinflammatory HS phenotype in macrophages by NKA inhibition. The addition of MAN together with OUA to induce hypertonic stress and Na^+^ accumulation in parallel resembled HS conditions better. The combination treatment increased *Nfat5* mRNA in a manner similar to HS levels and improved antimicrobial defense performance compared to controls. We conclude that intracellular Na^+^ availability and hypertonic stress together contribute to the induction of HS-enhanced macrophage activity.

## 6. Limitations of the Study

In our study, we show that the inhibition of NKA with the two CGs OUA and DIG induces intracellular Na^+^ accumulation in macrophages that resemble the HS environment. However, pharmacological inhibitors can cause side effects that also affect macrophage behavior. Therefore, complementary data with macrophages lacking NKA would help to investigate the role of NKA in Na^+^ sensing and control of macrophages further. However, the deletion of NKA is subject to further caveats. The introduction of an NKA knockout could alter macrophage viability, as NKA is known to be responsible for regulating membrane potential. Moreover, several isoforms of NKA are differentially expressed in different cell types. Presumably, transgenic macrophage cell lines or mouse models that allow inducible deletion of the specific NKA isoforms expressed by the cells would have to be used for this purpose.

## Data Availability

Original data are available on request from the corresponding author.

## Supplementary Materials

The following supporting information can be downloaded at: https://www.mdpi.com/article/10.3390/cells12242816/s1, Figure S1: Sketches of all the experimental setups applied. Figure S2: Intracellular Na^+^ levels after treatment with different concentrations of cardiac glycosides. Figure S3: Intracellular K^+^ levels after treatment with different concentrations of cardiac glycosides. Figure S4: Rb^+^ uptake assay confirms NKA-inhibition with cardiac glycosides. Figure S5: Treatment with cardiac glycosides or mannitol induces no cellular cytotoxicity nor impairs the growth of *E. coli*, and mannitol does not affect intracellular Na^+^ levels.
