# Supplementary material for: Simultaneous Increases in Intracellular Sodium and Tonicity Boost Antimicrobial Activity of Macrophages
Source: Cells. 2023 Dec 11;12(24):2816. doi: 10.3390/cells12242816 (PMC10741518; doi:10.3390/cells12242816)
Supplement: Supplementary file 1 [file cells-12-02816-s001.zip › cells-2614828-supplementary.pdf]

Supplementary Material

Figure 1a)-h), S1, S2

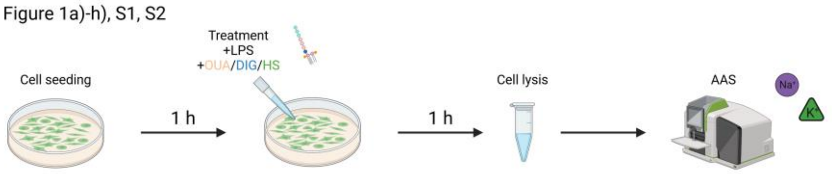

Figure 1i)-j)

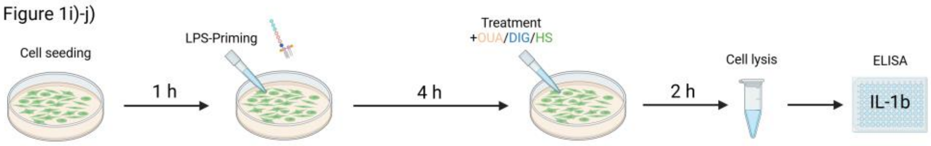

Figure 2a)-c)

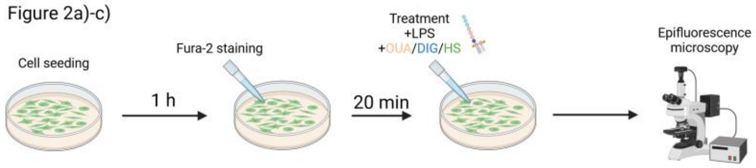

Figure 3a-c)

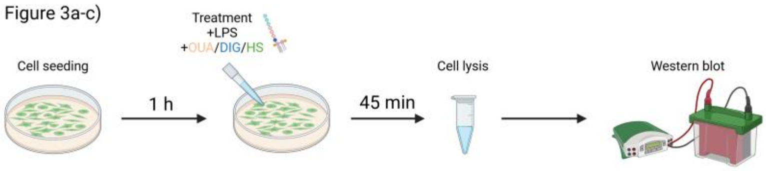

Figure 3d)-g)

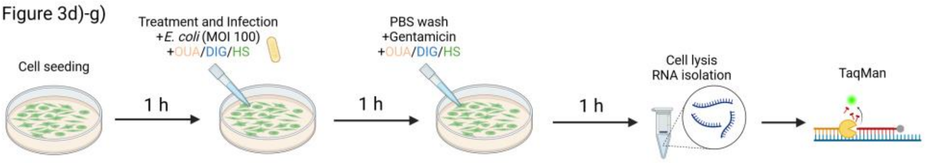

Figure 3h)-i)

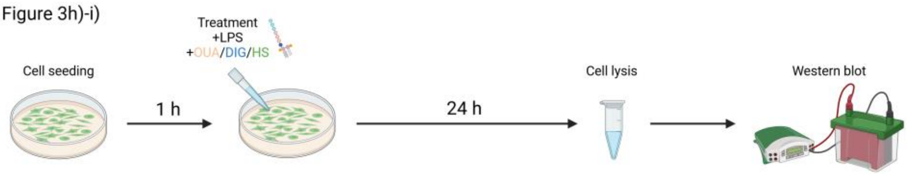

Figure 4a)-d), S4a)-d), S4h)

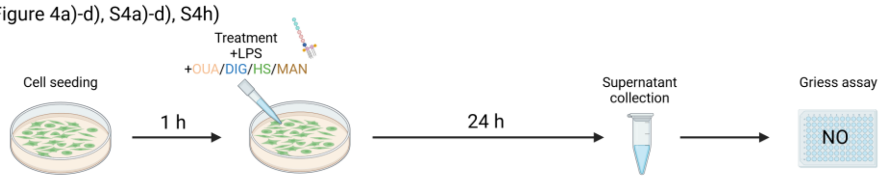

Figure 4e)

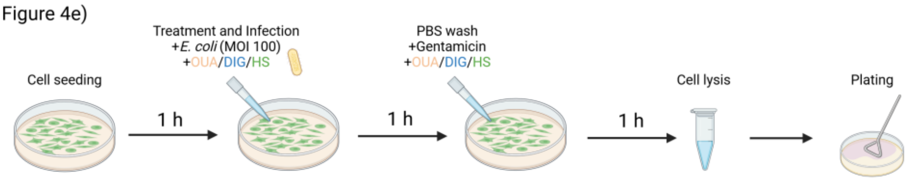

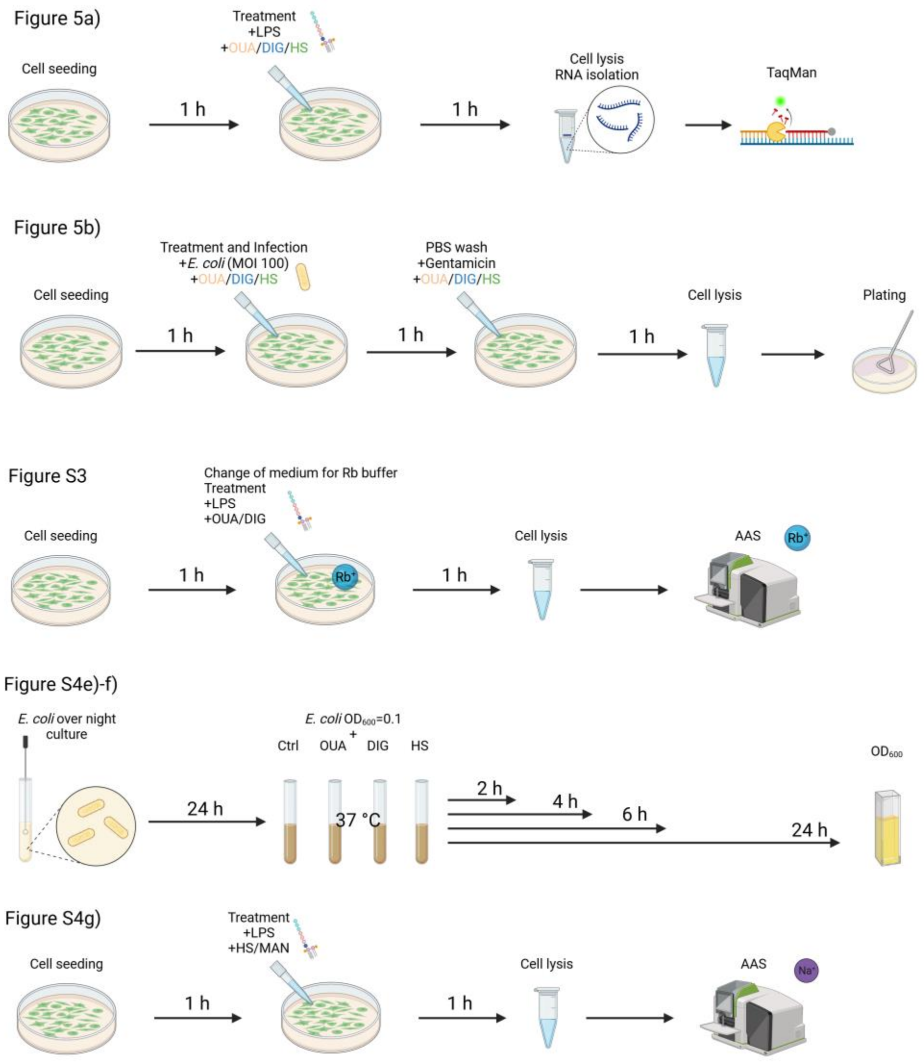

Figure S1: Sketches of all the experimental setups applied. Graphical illustrations of all the performed experiments of this study, displaying time points and types of treatments, as well as the applied analyses. Graphs were created with Biorender.

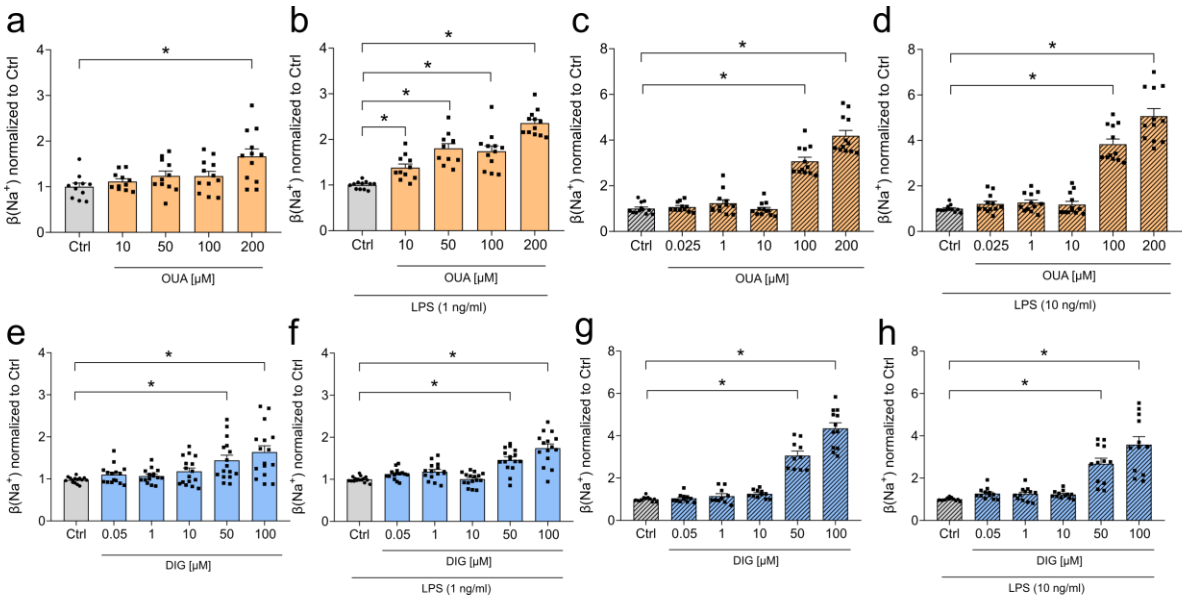

Figure S2. Intracellular Na^+ levels after treatment with different concentrations of cardiac glycosides**.** BMDMs (a/b/e/f) and RAWs (c/d/g/h; hatched graphs) were treated with different concentrations of (a/b/c/d) OUA and (e/f/g/h) DIG for one hour. (b/d/f/h) Cells were stimulated simultaneously with LPS. After cell lysis, $[Na^+]_i$ levels were determined and normalized to control conditions (Ctrl) (means ± s.e.m; n= 10-16; ordinary one-way ANOVA with Bonferroni's multiple comparisons test and Kruskal-Wallis test with Dunn's multiple comparisons test; *p < 0.05).

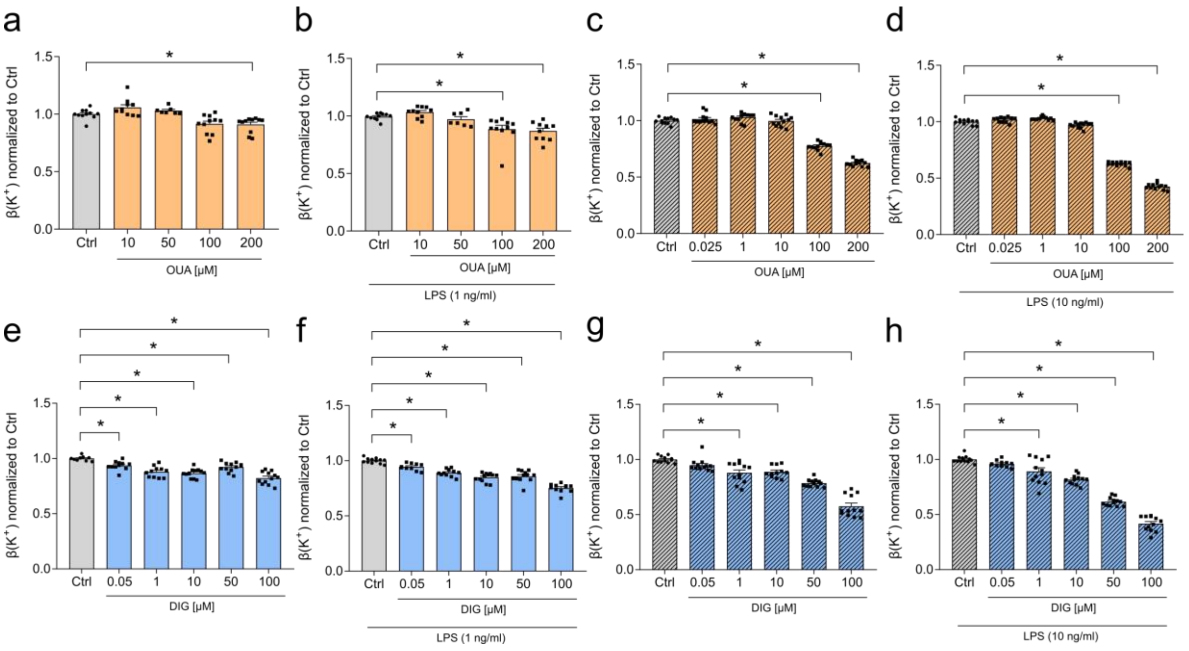

Figure S3. Intracellular K^+ levels after treatment with different concentrations of cardiac glycosides**.** BMDMs (a/b/e/f) and RAWs (c/d/g/h) were treated with different concentrations of (a/b/c/d) OUA and (e/f/g/h) DIG for one hour. (b/d/f/h) Cells were stimulated simultaneously with LPS. After cell lysis, $[K^+]_i$ levels were determined and normalized to control conditions (Ctrl) (means ± s.e.m; n = 7-12; Kruskal-Wallis test with Dunn's multiple comparisons test and ordinary one-way ANOVA with Bonferroni's multiple comparisons test; *p < 0.05).

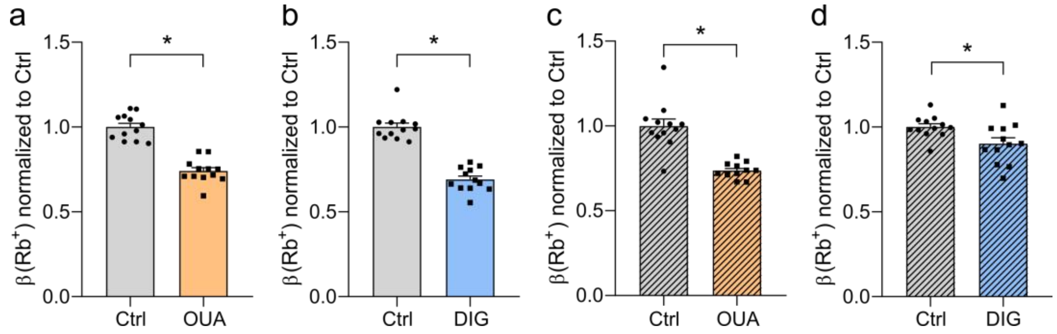

Figure S4. Rb^+ uptake assay confirms NKA-inhibition with cardiac glycosides. BMDMs (a/b) and RAWs (c/d) were incubated in Rb^+ uptake buffer and simultaneously treated with LPS and OUA or DIG. After one hour, macrophages were lysed and $[Rb^+]_i$ levels were determined and normalized to control conditions (Ctrl) (means ± s.e.m; n = 12; Unpaired t test with Welch's correction and Mann-Whitney test; *p < 0.05).

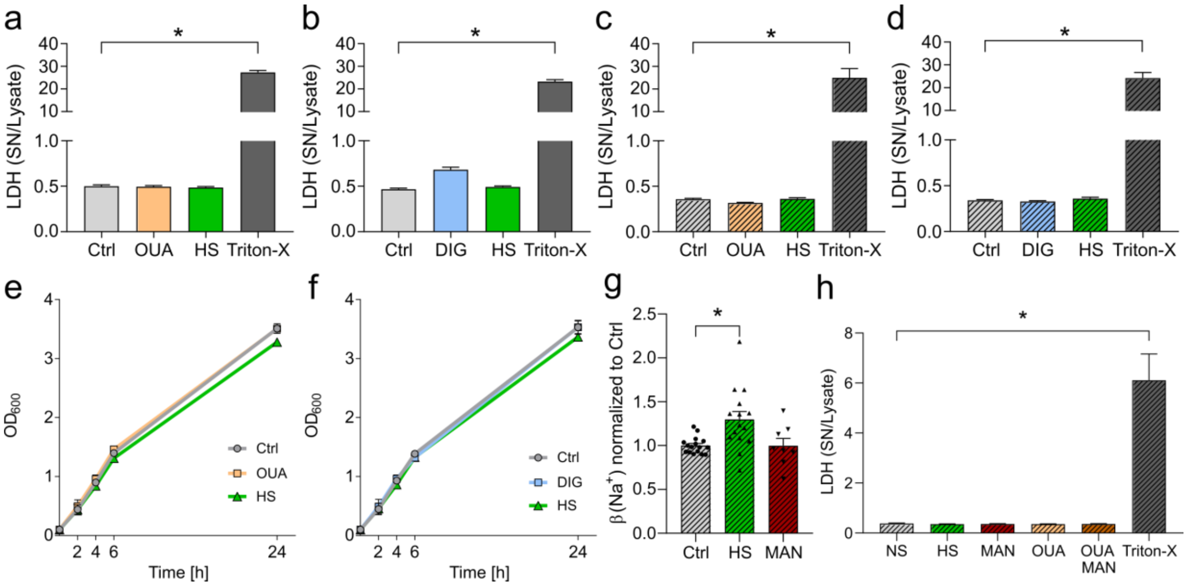

Figure S5: Treatment with cardiac glycosides or mannitol induces no cellular cytotoxicity nor impairs the growth of *E. coli*, and mannitol does not affect intracellular Na^+ levels. (a/b) BMDM were treated as in Fig. 4a/b. LDH ratios were determined. Triton-X was used as a positive control for cell death in every group (means ± s.e.m; n = 9-15; Ordinary one-way ANOVA with Bonferroni's multiple comparisons test; *p < 0.05). (c/d) RAWs were treated as in Fig. 4c/d. LDH ratios were determined. Triton-X was used as a positive control for cell death in every group (means ± s.e.m; n = 11-15; Kruskal-Wallis test with Dunn's multiple comparisons test; *p < 0.05). (e/f) *E. coli* were cultured in the absence of host cells in LB-media for 24 hours and treated with (e) OUA, (f) DIG or (e/f) HS. OD600 values were determined at indicated time points (means ± s.e.m; n = 6; two-way ANOVA with Geisser-Greenhouse correction; *p < 0.05). (g) RAWs were incubated with HS or MAN for one hour where indicated. Cells were stimulated simultaneously with LPS. After cell lysis, $[Na^+]_i$ levels were determined and normalized to Ctrl conditions (means ± s.e.m; n = 8 -16; ordinary one-way ANOVA; *p < 0.05). (h) RAW 264.7 macrophages were stimulated with LPS and simultaneously treated as indicated. After 24 h, LDH ratios were determined. Triton-X was used as a positive control for cell death in every group (means ± s.e.m; n = 8-20; Kruskal-Wallis test with Dunn's multiple comparisons test; *p < 0.05).
